# Putative cancer stem cells may be the key target to inhibit cancer cell repopulation between the intervals of chemoradiation in murine mesothelioma

**DOI:** 10.1186/s12885-018-4354-1

**Published:** 2018-04-27

**Authors:** Licun Wu, Walter Blum, Chang-Qi Zhu, Zhihong Yun, Laszlo Pecze, Mikihiro Kohno, Mei-Lin Chan, Yidan Zhao, Emanuela Felley-Bosco, Beat Schwaller, Marc de Perrot

**Affiliations:** 1 Division of Thoracic Surgery, Latner Thoracic Surgery Laboratories, University Health Network, Toronto, ON Canada; 2 Princess Margaret Cancer Centre, University Health Network, Toronto, ON Canada; 3 Department of Medicine, Unit of Anatomy, University of Fribourg, CH-1700 Fribourg, Switzerland; 4 INSERM, U1162, Génomique Fonctionnelle des Tumeurs Solides, 27 rue Juliette Dodu, 75010 Paris, France; 5 Laboratory of Molecular Oncology, University Hospital Zurich, University of Zurich, 8044 Zürich, Switzerland; 6 Division of Thoracic Surgery, Toronto Mesothelioma Research Program, Toronto General Hospital, 9N-961, 200 Elizabeth St, Toronto, ON M5G 2C4 Canada

**Keywords:** Mesothelioma stem cell (MSC), RN5-EOS-Puro2 cells, Cancer cell repopulation, Microarray, Chemoradiation

## Abstract

**Background:**

Cancer cell repopulation during chemotherapy or radiotherapy is a major factor limiting the efficacy of treatment. Cancer stem cells (CSC) may play critical roles during this process. We aim to demonstrate the role of mesothelioma stem cells (MSC) in treatment failure and eventually to design specific target therapies against MSC to improve the efficacy of treatment in malignant mesothelioma.

**Methods:**

Murine mesothelioma AB12 and RN5 cells were used to compare tumorigenicity in mice. The expression of CSC-associated genes was evaluated by quantitative real-time PCR in both cell lines treated with chemo-radiation. Stemness properties of MSC-enriched RN5-EOS-Puro2 cells were characterized with flow cytometry and immunostaining. A MSC-specific gene profile was screened by microarray assay and confirmed thereafter. Gene Ontology analysis of the selected genes was performed by GOMiner.

**Results:**

Tumor growth delay of murine mesothelioma AB12 cells was achieved after each cycle of cisplatin treatment, however, tumors grew back rapidly due to cancer cell repopulation between courses of chemotherapy. Strikingly, a 10-times lower number of irradiated cells in both cell lines led to a similar tumor incidence and growth rate as with untreated cells. The expression of CSC-associated genes such as CD24, CD133, CD90 and uPAR was dramatically up-regulated, while others did not change significantly after chemoradiation. Highly enriched MSC after selection with puromycin displayed an increasing GFP-positive population and showed typical properties of stemness. Comparatively, the proportion of MSC significantly increased after RN5-EOS parental cells were treated with either chemotherapy, γ-ray radiation, or a combination of the two, while MSC showed more resistance to the above treatments. A group of identified genes are most likely MSC-specific, and major pathways related to regulation of cell growth or apoptosis are involved. Upregulation of the gene transcripts *Tnfsf18*, *Serpinb9b*, *Ly6a*, and *Nppb* were confirmed.

**Conclusion:**

Putative MSC possess the property of stemness showing more resistance to chemoradiation, suggesting that MSC may play critical roles in cancer cell repopulation. Further identification of selected genes may be used to design novel target therapies against MSC, so as to eliminate cancer cell repopulation in mesothelioma.

**Electronic supplementary material:**

The online version of this article (10.1186/s12885-018-4354-1) contains supplementary material, which is available to authorized users.

## Background

Malignant pleural mesothelioma (MPM) is a highly aggressive malignancy with poor prognosis, most often associated with long-term exposure to asbestos [1]. However, limited efficacy of conventional chemotherapy, radiotherapy and surgery drives investigators to explore novel approaches to treat this disease [2–4].

Over the past decade, a number of clinical trials has been conducted looking at a tri-modality approach using induction chemotherapy, followed by surgery with an extrapleural pneumonectomy (EPP) and adjuvant hemi-thoracic radiation [5–7]. We have developed an innovative approach for MPM treatment with a short accelerated course of high-dose hemi-thoracic intensity-modulated radiation therapy followed by EPP. The initial phase I/II study assessed the feasibility of Surgery for Mesothelioma After Radiation Therapy (SMART). This innovative protocol SMART yields encouraging results and supports future studies looking at long-term outcome in patients with epithelioid MPM subtypes [8].

Here, we focus on immune modulation to trigger immune responses against mesothelioma stem cells (MSC) during the intervals of cytotoxic therapy. Evidence supports the notion that cancer cell repopulation can be attributed to cancer stem cells due to their higher resistance to conventional therapy and that this phenomenon could be inhibited or eliminated by a T cell response.

Our previous translational work showed that an antitumor effect through modulation of specific T cell responses may be achieved in murine model systems. For example, activation of natural killer T cells, depletion of regulatory T (Treg) cells, or systemic blockade of the immune checkpoint inhibitory regulator of T-cell immunity CTLA-4, between cycles of chemotherapy, was shown to inhibit cancer cell repopulation by enhancing specific antitumor immunity in murine mesothelioma [9–13]. We also demonstrated that anti-CTLA4 treatment can promote the abscopal effect to distant tumor sites after the primary tumor received local radiation [14]. Cancer cell repopulation can be inhibited or eliminated by T cell responses, suggesting that resistance to conventional therapy may be related to CSC escaping the immune system [15–20].

Thus, it would be beneficial to target MSC, if they could be unambiguously identified. However, the biggest challenge to target MSC is the lack of specific markers to identify them [21]. Since the MSC population is rather small among all tumor cells (estimated to be less than 5% in most cases), it remains a big hurdle to accumulate and isolate a large number of putative MSC, even if cell sorting can be used; the isolation procedure is time- and labour-consuming. Isolation of sphere-forming cells [22] has limitations as well, since those cells may initiate from CSC, but don’t necessarily have to be composed entirely of CSC [23].

The idea on how to achieve isolation of a large number of putative MSC comes from stem cell biology. The method was initially used to select induced pluripotent stem cells (iPS) with the help of an EOS (Early transposon Oct4 and Sox2 enhancer) system as a vector by selecting Sox2 and Oct4-expressing cells. The plasmid construct has Sox2- and Oct4-binding sites in the promoter region, which then drives GFP expression. The plasmid also contains a puromycin resistance gene that allows to identify subpopulations in the murine mesothelioma cell line RN5-EOS characterized by high levels of EOS reporter activity [24]. In the whole population of RN5-EOS cells approximately 5% of cells show GFP expression [25]. The selection with puromycin allows for the enrichment of the putative MSC named RN5-EOS-Puro2 [26]. Indeed, putative MSC are approximately 50–70% GFP-positive (GFP^+^) after 7 days of selection. Thus, we consider the high yield of putative MSC as a requirement for identification of stem cell markers and preparation of putative MSC lysates to design novel target therapies.

## Methods

### Cell lines

The murine mesothelioma cell line RN5 derived from C57Bl/6 mice was established recently by our team [27] and the cell line AB12 (from Balb/c mice) was kindly provided by Dr. Jay Kolls, University of Pittsburgh, Pittsburgh, PA. Both cell lines were maintained in RPMI 1640 medium supplemented with 10% fetal bovine serum and 1% penicillin and streptomycin, and maintained at 37 °C in an atmosphere containing 5% CO_2_. Cells were treated prophylactic with 5 μg/ml Plasmocin™ (Invivogen) for at least 2 weeks and were confirmed as mycoplasma-free. Cells were used for experiments at the time point of exponential growth (approximate 90% confluence) for all experiments.

**Fig. 1:**
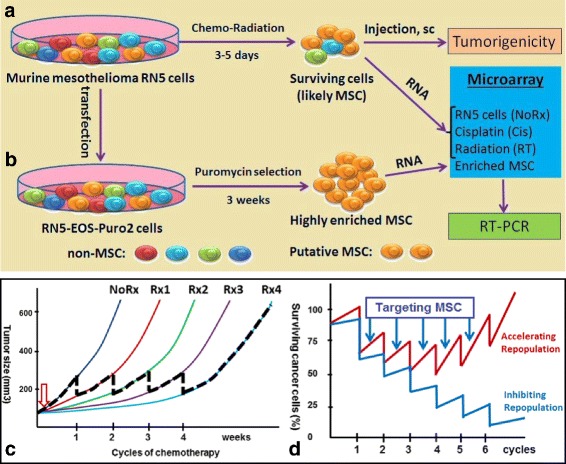
Experimental design and long-term goal of this study. Cancer cell repopulation during courses of chemotherapy and possibly mesothelioma stem cells (MSC) might play critical roles in tumor relapse. **a** Murine mesothelioma RN5 cells were treated with conventional therapy (cisplatin or γ-ray radiation) and surviving cells were collected for further experiments to evaluate tumorigenicity and gene expression profiles; **b** RN5 cells were transduced with lentivirus and selected with puromycin resulting in RN5-EOS-P2 cells enriched in MSC. Microarray analysis was performed to screen for gene profiles specific for MSC. **c** AB12 tumor growth in Balb/c mice after treatment with cisplatin once weekly, 5 μg/kg body weight, up to 4 cycles (Rx1–4) to mimic clinical settings (*n* = 5 mice). Tumor growth delay was achieved by each cycle of chemotherapy, however, tumors grew back rapidly due to cancer cell repopulation between courses of weekly chemotherapy. NoRx: no treatment, Rx1–4: number of doses of cisplatin; **d** Modeling the effects of specific MSC targeting: the model is based on the assumption that targeting MSC would delay/prevent tumor repopulation during the intervals of chemotherapy (blue curve) in comparison to conventional treatment (red curve) that mostly targets non-MSC

Details on the generation of RN5-EOS-Puro2 cells have been described before [25]. Briefly, the method was initially used to select for induced pluripotent stem cells (iPS) with the help of an EOS (Early transposon Oct4 and Sox2 enhancer) system as part of the vector. The plasmid construct has Sox2- and Oct4-binding sites in the promoter region, which then drives GFP expression and furthermore contains a puromycin resistance separated by an IRES. Puromycin (2 μg/ml; Life Technologies, China) was used for the selection process. This technique allowed us to identify the RN5 subpopulations characterized by high activity of the reporter, which was enriched for tumor initiating activity [24]. As the overall design in this study is depicted in Fig. 1, we compared these cells to murine mesothelioma RN5 cells treated with conventional therapy (cisplatin or γ-ray radiation), where surviving tumor cells were collected for further experiments to evaluate tumorigenicity and gene expression.

### Incidence of syngeneic mesothelioma cell-derived tumors in mice

RN5 and AB12 cells treated with γ-ray irradiation of 15Gy (10^4^ and 10^5^ cells, as indicated in the figure legend) were injected subcutaneously (s.c.) into the right flank of 6–8 week-old female C57BL/6 and Balb/c mice, respectively, both mouse strains purchased from The Jackson Laboratory (Bar Harbor, Maine), while the untreated parental cells (10^5^ and 10^6^ cells) were injected respectively into the right flank of other mice as controls. The tumor incidence and tumor growth curves were plotted according to the maximal perpendicular diameters as a function of days after tumor challenge. When tumors grew to the designated size (5 mm in diameter), the two maximal perpendicular diameters were measured with a calliper twice weekly to evaluate tumor incidence and growth. At the end points mice were sacrificed in accordance to the euthanasia guidelines of the Animal Use Protocol (AUP# 3399), which was approved by the Animal Resources Centre of University Health Network (UHN), Toronto, Canada.

### Treatment of cells and RNA extraction

Parental RN5-EOS cells and highly enriched MSC RN5-EOS-Puro2 cells (10^6^ cells/5 ml/well in a 6-well plate) were treated with cisplatin (Onco-Tain™ Hospira, UK) either with 0, 1 or 5 μg/ml overnight, or γ-ray radiation 5Gy and 15Gy by Cs-137 Gamma Cell Irradiator-40 (Atomic Energy of Canada Ltd., Ottawa, Canada) at a dose rate of approximately 100 cGy/min. Surviving cells were collected after overnight culture and used to extract RNA; dead floating cells and cell debris were washed away. The procedure of RNA extraction was performed according to the manufacturer’s instruction (QIAGEN, RNeasy Microarray Tissue Mini Kit, CA). Total RNA was treated with a Purelink DNase set (Thermo Fisher Sci., CA). RNA was used for cDNA synthesis using the Cloned AMV First-Strand cDNA Synthesis Kit (Thermo fisher Sci., CA). Gene expression was evaluated by real-time PCR or microarray assay.

### Real-time PCR

The oligos of previously reported CSC-associated genes and their oligo sequences (5′-3′) are composed as follows:

**Table:** 

Genes	Primer sequence 5′-3’
CD24	F CTGCTTCTGGCACTGCTCCTA
	R CGGTGCAACAGATGTTTGGT
CD34	F ACCCACCGAGCCATATGCT
	R CAGATACCCTGGGCCAACCT
CD68	F CTGCTCAGCTGCCTGACAAG
	R CCAATGATGAGAGGCAGCAA
uPAR	F GGCGACTACCTGTGTCCCA
	R CTCCTCTACCAGGCAGCTCTG
CD90	F ACCATGAACCCAGCCATCA
	R CTCGGGACACCTGCAAGACT
Bmi1	F TGATCAGAGCAGATTGGATCG
	R GCTGCTGGGCATCGTAAGT
CD117	F GCCAGTGCTTCCGTGACAT
	R GTCGTACGTCAGGATTTCTGGTT
CD133	F CGTGCTGGGAGGCAGAATA
	R GGGCATCCTTGGTCTGTTTG
Tnfsf18	F AAGGGCAGAGAGGTGCAAGAA
	R TGCAGGACTCGATGGCAGTT
Serpinb9b	F TATGGTCCTCCTGGGTGCAA
	R TGTCTGGCTTGTTCAGCTTCCT
Ly6a (Sca-1)	F CTGCCCCTACCCTGATGGA
	R GGGCAGATGGGTAAGCAAAGA
Nppb	F GGTGACACATATCTCAAGCTGCTTT
	R CAGCCAGGAGGTCTTCCTACAA

Quantitative PCR was performed according to a protocol previously reported [14].

Based on microarray-screened common genes, further confirmation of these genes was evaluated by real-time PCR. RN5 cells were treated with Cis 0.5 μg/ml, and 1.0 mg/ml, or RT 5Gy, 10Gy, and 15Gy, and harvested after 3 days to remove dead cells. RNA was extracted as stated before.

### Flow cytometry

RN5-EOS parental cells and RN5-EOS-Puro2 MSC were treated with cisplatin (1–5 μg/ml) overnight or/and γ-ray radiation 5Gy and 15Gy; cells were washed to remove dead cells and cell debris after overnight culture. All surviving cells were harvested and quantitated for the presence of GFP^+^ populations by flow cytometry. The presence of Tnfsf18, Ly6a (Sca-1), and CD90 was confirmed with staining of cells by monoclonal antibodies.

A Becton Dickinson LSR II Flow Cytometer (San Jose, CA) and FACS Diva™ software were used for analyses and data acquisition; FlowJo™ software was used for analysis.

### Immunofluorescence staining

MSC with or without treatment were harvested after washing with PBS and cultured in an 8-well Nunc® lab-Tek® chamber slide™ system (sigma-Aldrich) overnight. Slides were prepared for immunofluorescence staining with anti-GFP antibody conjugated with Alexa-488 and primary antibody rat anti-mouse β-actin (1:200); the secondary antibody anti-rat IgG-Alexa555 and DAPI (cell signaling) were applied following the manufacturer’s instructions. Fluorescence images of whole slides were captured by a Nikon inverse microscope (60× or 100×, NA 1.4, oil immersion objectives) connected with a Yokogawa spinning disk confocal system with Zeiss Axiovert 200 M inverted microscope (Gottingen, Germany). Images were acquired using the Volocity software (Waltham, MA).

### Microarray data analysis

RN5 cells growing in the exponential phase were harvested and treated in 6-well plates (10^6^ cells/5 ml/well). Four groups consisted of: 1) Parental RN5 cells without (no) treatment (NoRx), 2) RN5 cells treated with cisplatin (1 μg/ml) (Cis), 3) RN5 cells treated with γ-ray radiotherapy (5 Gy) (RT) and 4) RN5-EOS-Puro2 cells after puromycin selection to achieve enriched mesothelioma stem cells (MSC). Naïve peritoneum (N) was included as a negative control. Each group consisted of three samples. RNA was extracted as stated previously. Affymetrix Mouse Gene 2.0 microarray was performed to screen for potential mesothelioma or MSC-associated genes. Affymetrix Transcriptome Analysis Console (TAC) software (Santa Clara, CA) and Partek® Genomics Suite® software (St. Luis, MI) were used for data analysis. Genes with at least 2-fold changes were selected for further analysis. Comparisons among multiple groups were analyzed with ANOVA and differences were considered significant, if *P* values were less than 0.05.

Gene Ontology (GO) analysis was done using the GOMiner (https://discover.nci.nih.gov/gominer/htgm.jsp) web application. To expand the gene list of differentially expressed genes for a more stable gene ontology analysis, Pearson Correlation analysis (SAS v9.4, SAS Institute) was used to assess the correlated genes with the identified 41 genes and 0.98 < *r* < − 0.98 and *p* < 0.00001 were selected as the cutoffs.

### Overall experimental design and proposal of this study

Cancer cell repopulation during courses of chemotherapy and possibly MSC might play critical roles in tumor relapse. Murine mesothelioma RN5 cells were treated with conventional therapy (cisplatin or γ-ray radiation) and surviving cells were collected for further experiments to evaluate tumorigenicity and gene expression profiles (Fig. 1a); RN5 cells were transduced with lentivirus and selected with puromycin resulting in RN5-EOS-Puro2 cells enriched in MSC. Microarray analysis was performed to screen for gene profiles specific for MSC (Fig. 1b).

### Statistical analysis

All data are presented as the mean ± SEM. The comparison of gene expression and proportion of T cells between two groups was analyzed by using an unpaired Student’s *t* test. ANOVA was performed when compared among multiple groups using GraphPad Prism 6.0 statistical software (La Jolla, CA). A value of *P* < 0.05 was considered significantly different for all comparisons. * *P* < 0.05; ** *P* < 0.01; *** *P* < 0.001; **** *P* < 0.0001*.*

## Results

### Mesothelioma stem cells (MSC) may play a critical role in cancer cell repopulation during weekly cycles of chemotherapy and thus serve as a potential target

Murine mesothelioma AB12-derived tumors were treated with chemotherapy once weekly to mimic clinical settings. A tumor growth delay was achieved with each cycle of chemotherapy: however, tumors grew back rapidly due to cancer cell repopulation between the courses of weekly chemotherapy (Fig. 1c). Based on these results and considerable evidence reported previously, we proposed the hypothesis that targeting mesothelioma stem cells (MSC) might be able to inhibit cancer cell repopulation during the intervals of chemotherapy (Fig. 1d).

### Tumor incidence in syngeneic mice generated from surviving mesothelioma cells after γ-ray irradiation compared with tumors induced by untreated tumor cells

**Fig. 2:**
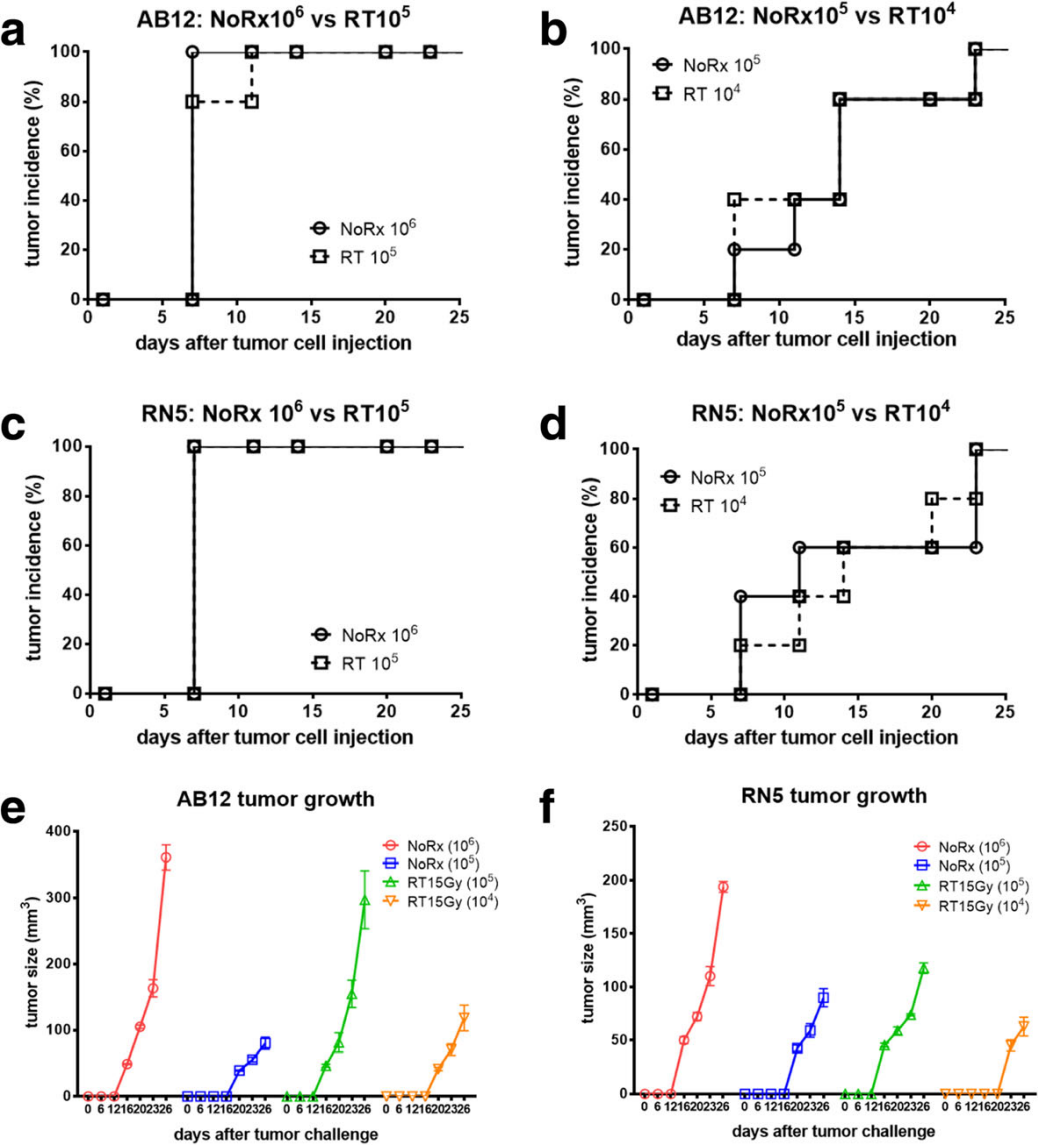
Tumor incidence in syngeneic mice resulting from injection of MM cells, either parental (no treatment; NoRx) or cells surviving γ-ray irradiation (RT). AB12 (**a**, **b**) and RN5 (**c**, **d**) cells were irradiated with 15 Gy γ-ray; surviving cells were used for s.c. injections in the right flank of mice (n = 5/group). The number of injected cells are indicated on the graphs. Surviving irradiated AB12 or RN5 cell injection resulted in the same tumor incidience with 10-fold less cells than when injecting the untreated AB12 or RN5 cells. The same results were obtained at two different doses of injected cells. **e**, **f** Start of tumor development was considered, when tumors become palpable; measurements of the two maximum perpendicular diameters were used to calculate the tumor size

Both murine mesothelioma AB12 and RN5 cells were irradiated with 15Gy γ-ray and surviving cells (3 days post-radiation) were injected into the respective syngeneic mice. Interestingly, the tumor incidence resulting from the injection of surviving irradiated AB12 (Fig. 2a & b) or RN5 cells (Fig. 2c & d) was of similar magnitude in comparison to the tumor incidence observed with a 10-fold higher load of the untreated (parental) cells. For instance, 1 × 10^5^ irradiated AB12 or RN5 cells resulted in a similar tumor incidence as with 1 × 10^6^ of untreated AB12 or RN5 cells (Fig. 2a & c); if the number of irradiated cells was decreased to 1 × 10^4^, the tumor incidence was quite similar to that observed after injection of 1 × 10^5^ untreated cells in both cell lines (Fig. 2b & d). Besides tumor incidence, also tumor growth (size determined at different time points) in mice after challenging with 10-times less of the irradiated cells was also quite similar as with the higher load of untreated cells (Fig. 2e & f, compare red and green curves, as well as blue and orange curves). Results with either AB12 or RN5 cells were nearly identical.

### Expression of CSC-associated genes was up-regulated in AB12 and RN5 cells after treatment with chemotherapy or radiation

Total RNA derived from surviving AB12 and RN5 cells after treatment with either cisplatin or γ-ray radiation was used to perform quantitative real-time PCR. Genes of interest were selected based on available literature previously reporting on genes likely associated with CSC in malignant mesothelioma. The treatments were the same as before: cisplatin (1 or 5 μg/ml) or γ-ray radiation (5 or 15 Gy). Some of the genes that were significantly upregulated, included CD24, CD90, CD68, CD117 in both cell lines, while other genes tended to be upregulated particularly in AB12 cells, while differences did not reach significance in RN5 cells (CD133, uPAR, CD34, Bmi-1). The genes CD24, CD117 and CD68 showed increased expression after either treatment (Additional file 1: Figure S1).

### Establishment of transduced RN5-EOS-Puro2 cells and confirmation of some stemness properties

**Fig. 3:**
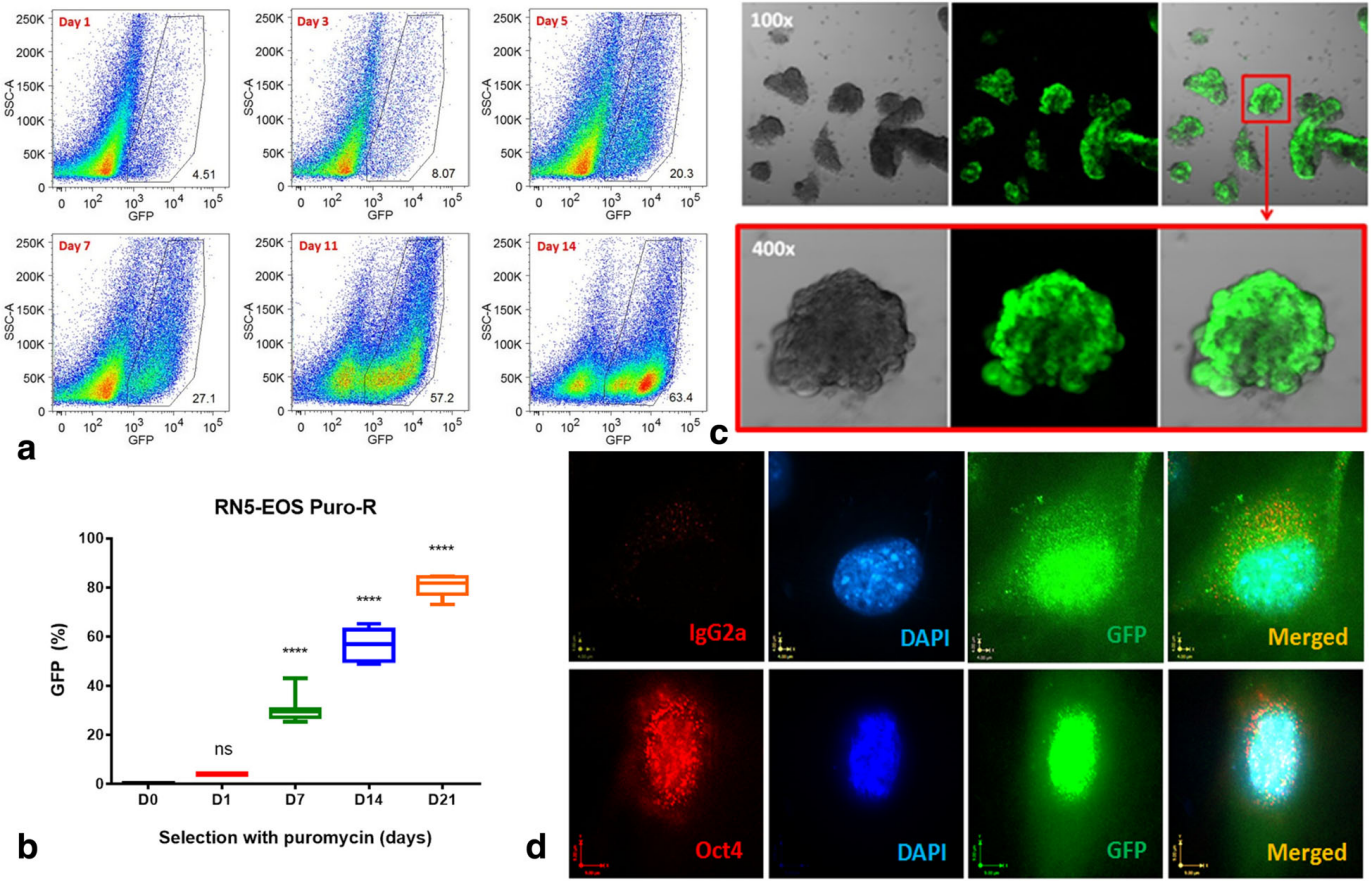
Characterization of transduced RN5-EOS-Puro2 cells and confirmation of properties of stemness. **a** Highly enriched MSC were obtained after 2 weeks of selection with puromycin; note the increase in the GFP-positive population over time reaching 63.4% after 14 days (right lower image). **b** Quantification of the MSC GFP^+^ population determined by flow cytometry from the beginning of puromycin selection (D0) up to D21**. c** Sphere-formation assay: highly enriched MSC from the above selection were cultured in ultra-low adherent 24-well plates and spheres were formed in approximately 7–10 days. **d** Highly enriched MSC were stained for Oct4, which was positive in the nucleus (oil immersion objective; 60×)

**Fig. 4:**
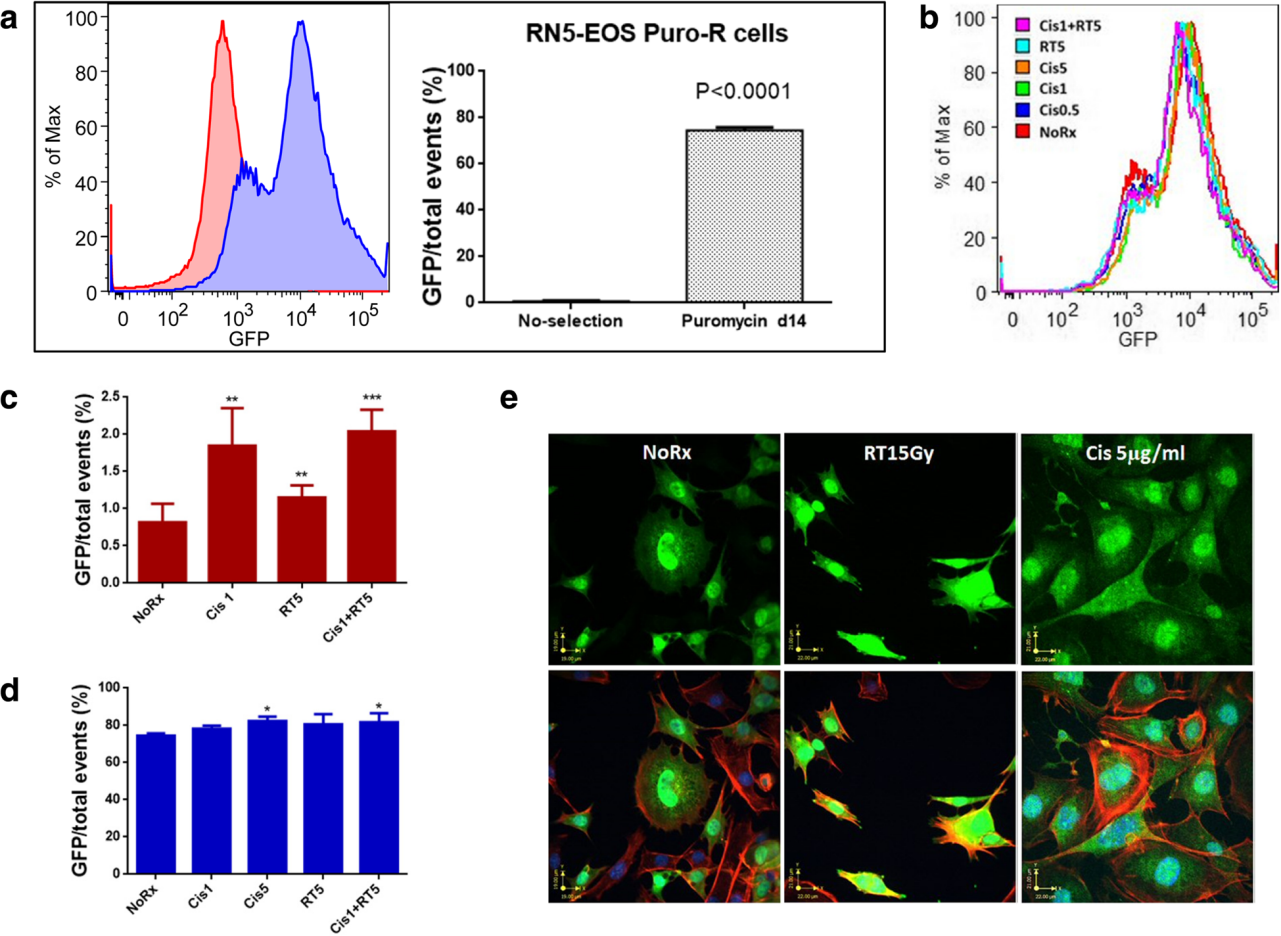
Mesothelioma stem cells are more resistant to chemotherapy or radiation treatment in vitro*.* **a** The majority of highly enriched RN5-EOS-Puro2 cells (2 μg/ml for 14 days) were GFP-positive as shown in the histograms (blue curve) if compared with the unselected parental RN5-EOS cells (red curve). **b** RN5-EOS-Puro2 cells were treated with cisplatin or γ-ray radiation and were more resistant. **c**, **d** Untreated parental RN5-EOS cells and RN5-EOS-Puro2 cells were treated with cisplatin or γ-ray radiation. Cisplatin and radiation resulted in significant increases of the GFP-positive cell populations; in the selected MSC-enriched population, only cisplatin (alone or in combination with irradiation) slightly increased the fraction of GFP^+^ cells **e**) RN5-EOS-Puro2 cells express high levels of GFP (green), even after treatment with 15 Gy γ-ray (RT15Gy) or cisplatin (Cis) 5 μg/ml, almost similar as is observed in RN5-EOS-Puro2 cells not subjected to any treatment. Actin immunoflurescence is shown for the outline of cells (red) and DAPI was used to stain nuclei (blue). A merged image is shown in the right lower corner

RN5-EOS-Puro2 cells were generated as reported before using a lentiviral plasmid construct [25]. Transduced RN5-EOS-Puro2 cells subjected to selection with puromycin for 2 weeks yielded a cell subpopulation highly enriched in GFP-positive (GFP^+^) cells assuming putative MSC (Fig. 3a); the gradual increase of GFP^+^ cells over time is demonstrated by FACS as shown in Fig. 4b. These highly-enriched MSC were obtained after puromycin treatment for at least 2–3 weeks; then the fraction of the strongly green GFP^+^ population reached approximately 65–85% as quantified by FACS (Fig. 3b). Serial numbers of puromycin-selected cells (50, 100 and 500 cells/well) were grown in ultra-low adherent 24-well plates; 3D spheres (mesospheres) were formed at approximately 7 days of culture consisting of cells with strong GFP fluorescence (Fig. 3c). Highly enriched MSC GFP^+^ cells were fixed and immunostained for Oct4. Positive staining for Oct4 was observed in the nucleus (Fig. 3d).

### Mesothelioma stem cells (MSC) are more resistant to chemotherapy or radiation treatment in vitro

A majority of the highly enriched MSC RN5-EOS-Puro2 cells after puromycin selection for 14 days were GFP-positive (as shown in the histogram as the blue population) compared with unselected RN5-EOS parental cells (red population) (Fig. 4a). Treatment with cisplatin or γ-ray radiation did not result in a dramatic change in the highly enriched MSC even though when given at higher doses of cisplatin (5 μg/ml) or in combination with γ-ray radiation RT5Gy, suggesting that they were more resistant to chemoradiation (Fig. 4b). In line with the presumed increased chemo- and/or radiation resistance of GFP^+^ cells, the fraction of green cells increased in the parental RN5-EOS population subjected to cisplatin (1 μg/ml), radiation (5Gy) or both (Fig. 4c). Under the selected conditions, it was evident that cisplatin had a stronger effect in sparing the GFP^+^ cells. A similar experiment carried out with RN5-EOS-Puro2 cells consisting of mostly green cells gave essentially identical results; however the magnitude of the effect was much smaller, since most cells consisted of already resistant cells. Only in the groups treated with cisplatin, a decrease of non-green cells, i.e., a higher fraction of GFP^+^ cells was evident (Fig. 4d). We also compared the morphology of the highly enriched MSC with or without treatment by confocal microscopy. GFP fluorescence signals were similar in untreated MSC (NoRx) in comparison to MSC after treatment with γ-ray radiation 15Gy (RT15Gy) or cisplatin (Cis) 5 μg/ml. This indicates that radiation has no direct effect on expression levels or intracellular localisation of GFP (Fig. 4e).

### Gene profile associated with mesothelioma and mesothelioma stem cells (MSC)

**Fig. 5:**
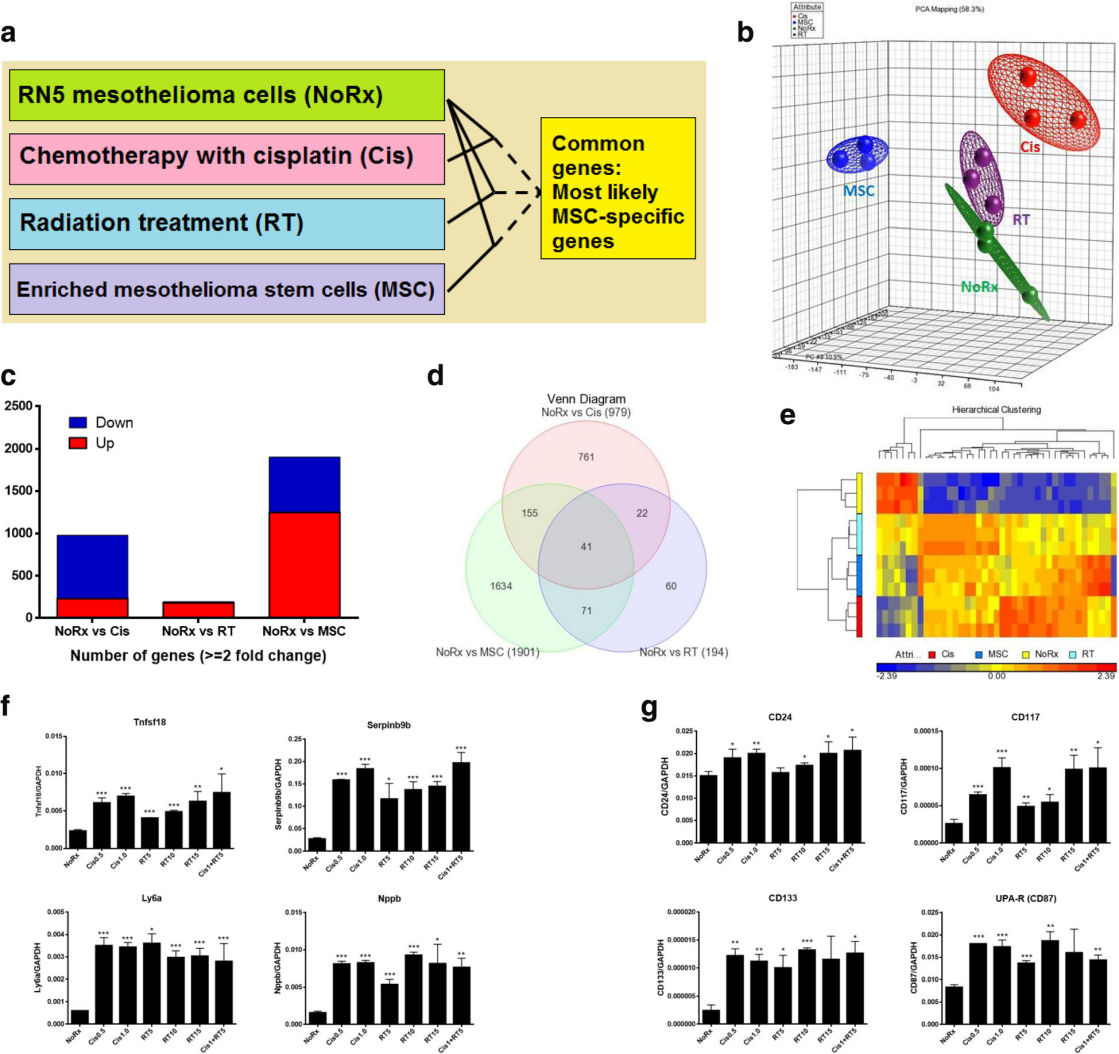
Mesothelioma stem cell-associated genes. **a** Screening strategy of mesothelioma stem cell-associated genes by comparing parental RN5 cells with no treatment (NoRx), with cisplatin (Cis), γ-ray radiotherapy (RT), and enriched mesothelioma stem cells (MSC); **b** Overall differentiation of gene expression determined by principal component assay (PCA) mapping; **c** Total number of genes with a greater than 2-fold change of up- or down-regulation; **d** Venn diagram showing the overlapping genes of the 3 comparisons as depicted in (**a**); **e** Heatmap of gene expression in the 4 groups (NoRx, Cis, RT and MSC) as screened in the Venn diagram, which most likely contains mesothelioma-associated stem cell genes; **f** Novel genes including Tnfsf18, Serpinb9b, Ly6a and Nppb are confirmed to be upregulated by RT-qPCR; **g** Known genes CD24, CD117, CD133 and uPAR (CD87) are upregulated, as well as after treatment with chemoradiation. The experiment was carried out twice. The ratio of each gene to the house-keeping gene GAPDH is presented as mean ± SD. * *P* < 0.05, **P < 0.01, ***P < 0.001

With the aim of finding differentially expressed genes (gene signatures) for different types of cells of mesothelial origin and for different treatments, a gene screening strategy was developed by comparing various groups (NoRx, Cis, RT and MSC) as depicted in Fig. 5a. Overall differences of gene expression were determined by principal component assay (PCA) mapping, which separated very well, indicating significant differences between groups (Fig. 5b). The number of genes with at least a 2-fold change and *p* values less than 0.05 including both up- and down-regulated genes among the 4 groups is shown in the bar graph and Venn diagrams (Fig. 5c & d). The largest difference (1901 genes) in gene expression levels was observed between the parental untreated RN5 cells and highly MSC-enriched RN5-EOS-Puro2 cells. The gene difference likely attributed to the critical genes of tumor cells and stem cells may by potential MSC-associated genes. Based on the finding that CSC are more resistant to cisplatin or γ-ray radiation, one would expect to observe an increase in MSC-state cells; the overlap between NoRx and Cis consisted of 761 genes and between NoRx and RT of 194, and the common genes of all three comparisons among NoRx, Cis, RT and MSC groups were narrowed down to 41 genes (Fig. 5d and Additional file 1: Table S1). A Heatmap of screened genes from the overlapping list in the Venn diagram highlights most likely MSC-associated genes. Two contrary clusters contain the up-regulated and down-regulated genes in MSC or after treatment with chemoradiation of RN5 cells compared with parental RN5 cells (Fig. 5e).

This group of selected genes is most likely associated with the MSC state. We hypothesize that among these genes, one or several might evolve as a biomarker to identify MSC.

### Confirmation of the genes of interest by RT-qPCR and flow cytometry

Confirmation of these genes was determined by quantitative real-time PCR. Gene expression of Tnfsf18, Serpinb9b, NPPB and Ly6a (Sca-1) were found upregulated significantly after treatment with Cis 0.5 μg/ml, 1 μg/ml or γ-ray radiation 5Gy, 10Gy, 10Gy or in combination of Cis 1 μg/ml and RT5Gy (Fig. 5f). Other genes including CD24, CD117, uPAR, and CD133 that were reported previously in mesothelioma were upregulated as well (Fig. 5g).

Preliminary studies have indicated that positive staining for Tnfsf18 and Ly6a was observed in RN5-EOS-Puro2 cells. Peritoneal lavage cells after i.p. injection of RN5 cells stained Tnfsf18 and Sca-1 double-positive (Additional file 1: Figure S2 *and unpublished data*).

The 41 common genes were screened by comparing the groups NoRx vs Cis, RT and MSC (Additional file 1: Table S1). The upregulated and downregulated genes were separated in a first step. The downregulated gene list was then dropped due to the low number, therefore, we concentrated on upregulated genes. When submitted to gene signature profiling (GSEA) and after ID conversion, Sca-1 (Ly-6a) was not present in the list of human genes and thus it does not appear in Additional file 1: Table S2. The remaining overlapping genes are shown in Additional file 1: Table S2, where we found that genes upregulated by k-ras are significantly increased.

**Fig. 6:**
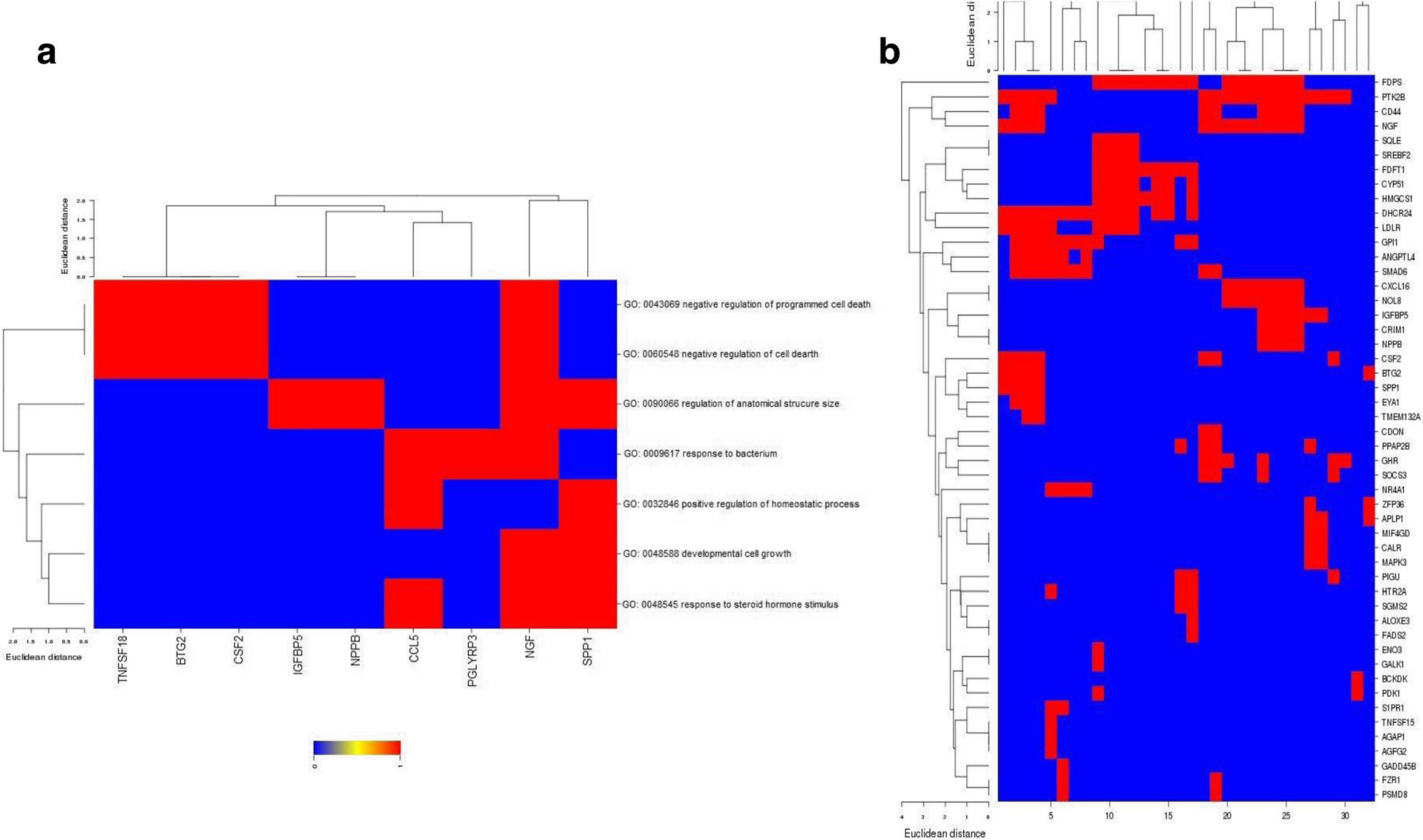
Gene ontology (GO) analysis of the identified genes. **a** After performing Pearson correlation analysis at cutoffs of 0.98 < *r* < − 0.98 and *p* < 0.00001, the 41 selected genes were expanded to 221 genes, which were identified to be significantly correlated with one of the 41 genes. These genes were found to be involved in as many as 32 pathways; **b** Seven pathways were identified at the cutoff of FDR < 0.05, and NGF and Spp1 are likely to be the dominant drivers of all these pathways

To evaluate the potential signaling pathways that these genes may be involved in, we first used the identified 41 genes to perform the gene ontology (GO) analysis (Table 1). At the cutoff of FDR < 0.05, 7 pathways related to positive regulation of cell development, differentiation, growth and negative regulation of cell death and apoptosis were identified and NGF and Spp1 may be the dominant drivers of all these pathways (Fig. 6a). To obtain a more stable conclusion of the GO analysis, we expanded the gene list by doing Pearson correlations of the 41 genes with the rest of the genes. At cutoffs of 0.98 < *r* < − 0.98 and *p* < 0.00001, 221 genes were identified to be significantly correlated with one of the 41 genes. GO analysis confirmed the involvement of pathways related to positive regulation of cell development, differentiation, growth and negative regulation of cell death and apoptosis. Again, NGF and Spp1 play a crucial role in these signaling pathways. In addition, CD44 and PTK2B genes that are closely correlated with NGF signaling were also identified (Fig. 6b and Additional file 1: Table S3).

**Table 1:** After expansion by Pearson, the 41 genes correlated with the rest 211 genes and involved in 32 pathways

Number	Pathway ID	Pathway	FDR^a^
1	GO:0008299	Isoprenoid biosynthetic process	0.002134
2	GO:0051346	Negative regulation of hydrolase activity	0.002385
3	GO:0006644	Phospholipid metabolic process	0.003211
4	GO:0006916	Anti-apoptosis	0.00327
5	GO:0043086	Negative regulation of catalytic activity	0.003652
6	GO:0042976	Activation of Janus kinase activity	0.004027
7	GO:0018106	Peptidyl-histidine phosphorylation	0.004027
8	GO:0006417	Regulation of translation	0.006019
9	GO:0031399	Regulation of protein modification process	0.006455
10	GO:0010608	Posttranscriptional regulation of gene expression	0.006471
11	GO:0031124	mRNA 3'-end processing	0.006525
12	GO:0040008	Regulation of growth	0.007398
13	GO:0008202	Steroid metabolic process	0.007429
14	GO:0008361	Regulation of cell size	0.007517
15	GO:0045793	Positive regulation of cell size	0.007545
16	GO:0045927	Positive regulation of growth	0.007662
17	GO:0006066	Alcohol metabolic process	0.007722
18	GO:0001932	Regulation of protein phosphorylation	0.00775
19	GO:0043154	Negative regulation of caspase activity	0.007818
20	GO:0016049	Cell growth	0.007857
21	GO:0043066	Negative regulation of apoptosis	0.007891
22	GO:0008610	Lipid biosynthetic process	0.007913
23	GO:0030307	Positive regulation of cell growth	0.008
24	GO:0007259	JAK-STAT cascade	0.008302
25	GO:0060548	Negative regulation of cell death	0.009413
26	GO:0001558	Regulation of cell growth	0.00978
27	GO:0043069	Negative regulation of programmed cell death	0.009786
28	GO:0051336	Regulation of hydrolase activity	0.009791
29	GO:0016126	Sterol biosynthetic process	0.01
30	GO:0008203	Cholesterol metabolic process	0.01
31	GO:0006695	Cholesterol biosynthetic process	0.01
32	GO:0016125	Sterol metabolic process	0.01

At cutoffs of 0.98<r<-0.98 and *p*<0.00001, 221 genes were identified to be significantly correlated with one of the 41 genes. ^a^*FDR* False discovery rate. FDR<0.01

## Discussion

Current therapies for MPM include cisplatin and pemetrexed-based chemotherapy, hemithoracic fractionated radiation, and surgery [28–30]. However, the efficacy is far from satisfying, even though some improvements have been achieved, and immunotherapy is probably the most promising strategy to control this disease through modulation of the immune response [31]. For instance, systemic blockade of immune checkpoints such as CTLA-4, PD-1 and its ligands PD-L1/2 has shown significant antitumor effects in certain cancers including mesothelioma [11, 32]. In our experience, we found that EPP performed after a short course of high dose hemithoracic radiation resulted in better outcome for the patients with an epithelioid MM subtype. Evidence in our laboratory has shown that the benefit is very likely due to the activation of a specific immune response against the tumor by the high dose radiation [8, 14, 33].

Cancer cells that are not killed by conventional cytotoxic therapy will repopulate the tumor and lead to treatment resistance [34]. Repopulation between treatments is likely one of the mechanisms to explain why some cancers respond initially to chemotherapy, but become resistant to continued treatment [35]. Therefore, selective inhibition of cancer cell repopulation might overcome drug resistance. Our results provide evidence that the proportion of MSC increases after chemoradiation, and that MSC are more resistant to the treatment of chemoradiation in murine mesothelioma. CSC may play critical roles in the process of cancer cell repopulation, however, biomarkers to identify CSC remain elusive [36–38]. Until recently the transcription factors Oct4, Sox2, and Nanog have been considered playing essential roles in early development and for the propagation of undifferentiated embryonic stem cells (ESC) in culture [39]. Recent results  provide new insights into the transcriptional regulation of stem cells and reveal how Oct4, Sox2, and Nanog contribute to pluripotency and self-renewal [40]. A new technique was developed to transduce transcription factor target genes into fibroblasts, so as to transform the property of fibroblasts to induced pluripotent stem cells (iPS) [41]. More strikingly, iPS reporter cell lines were generated for the identification and selection of pluripotent stem cells in vitro [24, 42]. Based on the above progress, we developed the RN5-EOS murine cell line, by transducing the EOS into RN5 cells with lentiviral vector. RN5-EOS cells with a GFP reporter can be selected by puromycin, so as to achieve a highly enriched population of stem cells. RN5 is a murine mesothelioma cell line, which was recently established after exposure to asbestos fibers in a C57BL/6 background mouse in our laboratory [27].

Due to availability of highly enriched MSC, it would be beneficial to design novel strategies targeting MSC. However, identification of MSC is still a big challenge [21]. By using microarray analysis, we compared MSC with RN5 parental cells after treatment with chemoradiation and found a group of genes that are most likely MSC-specific genes. In future studies, the functions of these genes need to be confirmed. Only a few genes such as *nppb* have been reported to play a critical role in ESC signaling pathways [43]. Another gene *Ly6a,* also known as *Sca-1,* has been identified as a CSC marker [44]*.* Other genes have not yet been demonstrated to be associated with stem cell properties.

Our preliminary results showed that tumor-specific T cells can be activated by co-culturing splenocytes derived from RN5-bearing mice after pulsation with whole cell lysates of parental RN5 or MSC-enriched RN5-EOS-Puro2 cells. We found that splenocytes derived from RN5 tumor-bearing mice had a significantly higher rate of T cell proliferation (identified by Ki67 staining) after pulsation of lysates (*p* = 0.0001). More encouragingly, RN5-EOS-Puro2 cell lysate resulted in a similar response of T cell proliferation with pan-antigens of RN5 cell lysate (*p* = 0.0224) (Additional file 1: Figure S3). It is required to identify MSC-specific markers.

Availability of highly enriched MSC made it possible to prepare a whole MSC lysate vaccine to enhance specific immunity against mesothelioma cells. Immune therapy targeting MSC in combination with conventional cytotoxic therapy is assumed to potentially improve the efficacy of treatment against this disease. This approach would open new avenues to mesothelioma treatment and also be feasible for other types of cancer.

In the future, we expect to combine this approach with removing the brake of immune checkpoints, to maximally boost the immune response to target MSC, and eventually to improve the efficacy of mesothelioma treatment. Evidence in our AB12 mesothelioma model indicated that systemic blockade of the immune checkpoints CTLA-4 and PD-1 in combination with chemo- or radiation therapy did result in tumor growth delay through enhancing antitumor immunity, such as activating T cells and decreasing Treg [14]. However, only few studies looked at the impact of CSC in mesothelioma. Programmed DC vaccine with MSC-specific peptides or whole cell lysates might have a powerful impact on MSC, since MSC are resistant to chemotherapy or radiation, but less resistant to immunotherapy.

In vitro assay of tumor-specific immune responses showed that after overnight co-culture of splenocytes derived from RN5-bearing mouse spleen pulsed with or without RN5 cell lysate or enriched RN5-EOS-Puro2 cell lysate, tumor specific T cell activation and proliferation were found remarkably increased compared with naïve splenocytes, suggesting that specific anti-tumor immune response can be enhanced by pulsation with both mesothelioma cell lysate and MSC whole cell lysate. If MSC markers could be confirmed, we would be able to monitor specific immunity against MSC and to design specific immunotherapy targeted against MSC.

The first DC vaccine was approved by the FDA in 2014 [20]. Intratumoral delivery of a DC vaccine was confirmed to be effective, especially after the tumor was treated with chemotherapy or radiation. Tumor-associated antigens (TAA) released from dead tumor cells are captured and processed by DC, and presented to naïve T cells. Activated T cells start migrating and trafficking to recognize tumor cells and eliminate them [45]. Immature DCs become mature after uptake of TAA. Mature DCs also release cytokines including IL-12 and IFN-γ that are able to kill tumor cells directly or indirectly by inhibiting tumor angiogenesis [46, 47]. A recent study had evaluated the specificity of DCs for breast cancer stem cells (BCSCs) in vitro and in vivo, and immature DCs were primed with BCSC-derived antigens to generate mature DCs. Modified DCs may be a promising therapy for treating drug-resistant cancer cells as well as CSC [48]. Also, MSC-associated genes could be introduced or eliminated to demonstrate their roles in maintaining stemness by genetic manipulation using the CRISPR technique [49].

MSC-specific markers also make it possible to design novel target therapies against MSC by neutralization with monoclonal antibodies. More promisingly chimeric antigen receptor T cells (CAR-T) may be modulated by using MSC-specific antigens [50]. Functional assays could be performed through manipulating individual genes.

## Conclusions

In conclusion, by further verifying the MSC-specific markers based on the group of genes screened by microarray, target therapy or specific immunotherapy may be designed thereafter. The enriched MSC can be used to prepare whole cell MSC lysate for vaccine alone or pulsation of DC derived from bone marrow. More interestingly, specific immune responses could be monitored, once MSC markers are determined. This novel DC vaccine would be able to activate T cells that specifically recognize MSC and eliminate them. It might be promising to focus on targeting MSC through immune modulation by specific T cell response in murine mesothelioma models. This work opens a new avenue for mesothelioma treatment as well as for other types of cancer.

## Additional file

Additional file 1: **Figure S1.** Expression of CSC-associated genes up-regulated in AB12 and RN5 cells after cisplatin treatment or radiation. **Table S1.** The selected gene list with 2 or more fold change in comparison with untreated RN5 cells. **Figure S2.** Tumor-associated macrophages may share the property of mesothelioma stem cells. **Table S2.** Selected genes of potential murine mesothelioma stem cells overlapped with human gene signature profiling (GSEA). **Table S3.** GO signaling pathways associated with the selected 41 genes. **Figure S3.** In vitro assay of tumor-specific T cell proliferation determined by flow cytometry. (PDF 666 kb)

## Abbreviations

Cis: Cisplatin
CSC: Cancer stem cells
EPP: Extrapleural pneumonectomy
GO: Gene Ontology
iPS: induced pluripotent stem cells
MPM: Malignant pleural mesothelioma
MSC: Mesothelioma stem cells
N: Naïve peritoneum
NoRx: No treatment
RT: Radiotherapy
TAC: Transcriptome Analysis Console
Treg: Regulatory T cell
