# Supplementary material for: Putative cancer stem cells may be the key target to inhibit cancer cell repopulation between the intervals of chemoradiation in murine mesothelioma
Source: BMC Cancer. 2018 Apr 27;18:471. doi: 10.1186/s12885-018-4354-1 (PMC5921988; doi:10.1186/s12885-018-4354-1)
Supplement: Supplementary file 1 — Figure S1. Expression of CSC-associated genes up-regulated in AB12 and RN5 cells after cisplatin treatment or radiation. Table S1. The selected gene list with 2 or more fold change in comparison with untreated RN5 cells. Figure S2. Tumor-associated macrophages may share the property of mesothelioma stem cells. Table S2. Selected genes of potential murine mesothelioma stem cells overlapped with human gene signature profiling (GSEA). Table S3. GO signaling pathways associated with the selected 41 genes. Figure S3. In vitro assay of tumor-specific T cell proliferation determined by flow cytometry. (PDF 666 kb) [file 12885_2018_4354_MOESM1_ESM.pdf]

## Supplementary data

***Supplementary figure 1. Expression of CSC-associated genes up-regulated in AB12 and RN5 cells after cisplatin treatment or radiation.*** *Both AB12 and RN5 cells (5x10$^6$ cells/petri dish) were treated with cisplatin (1µg/ml and 5ug/ml) or with γ-ray radiation (5 or 15 Gy). Total RNA was isolated for qRT-PCR. Genes of interest were selected based on the literature to determine genes likely associated with mesothelioma stem cells. A) AB12 cells, B) RN5 cells. Data were analyzed from 3 independent experiments. The ratio of each gene to the house-keeping gene GAPDH is reported as mean ± SD. * P<0.05, **P<0.01, ***P<0.001.*

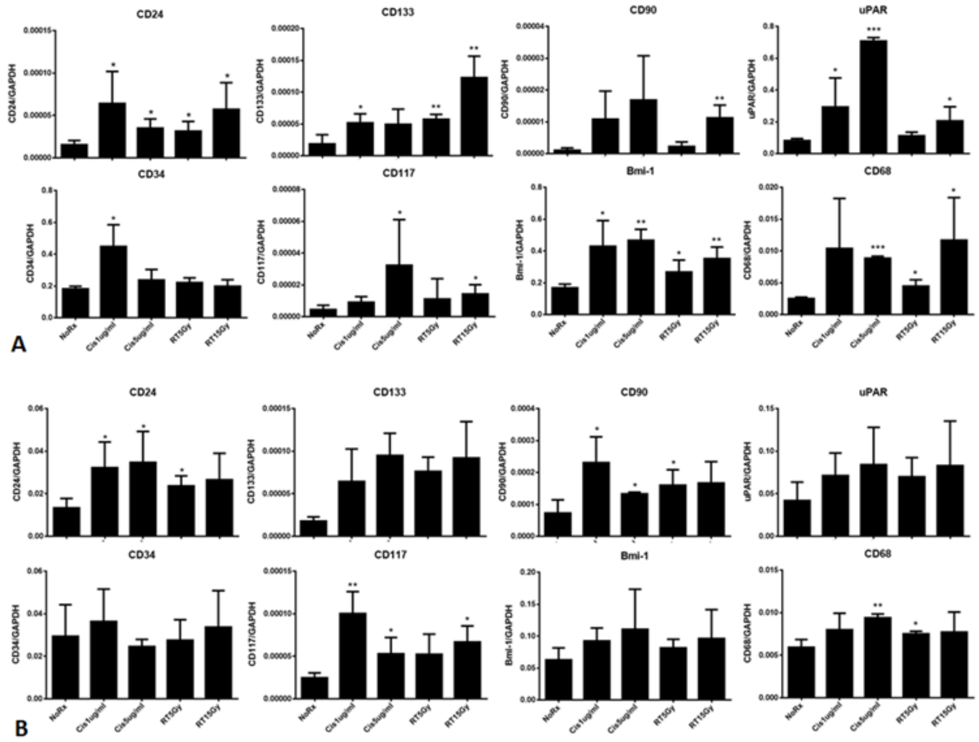

***Supplementary figure 2. Tumor-associated macrophages may share the common properties with mesothelioma stem cells.*** *A) Peritoneal lavage cells derived from mice at 4 weeks after i.p. injection of 4×10$^6$ RN5 cells, express high level of pan macrophage marker F4/80, Tnfsf18 and Sca-1(Ly6a) determined by flow cytometry; B) Both RN5-EOS-Puro2 cells and peritoneal lavage cells express high levels of F4/80 and Tnfsf18.*

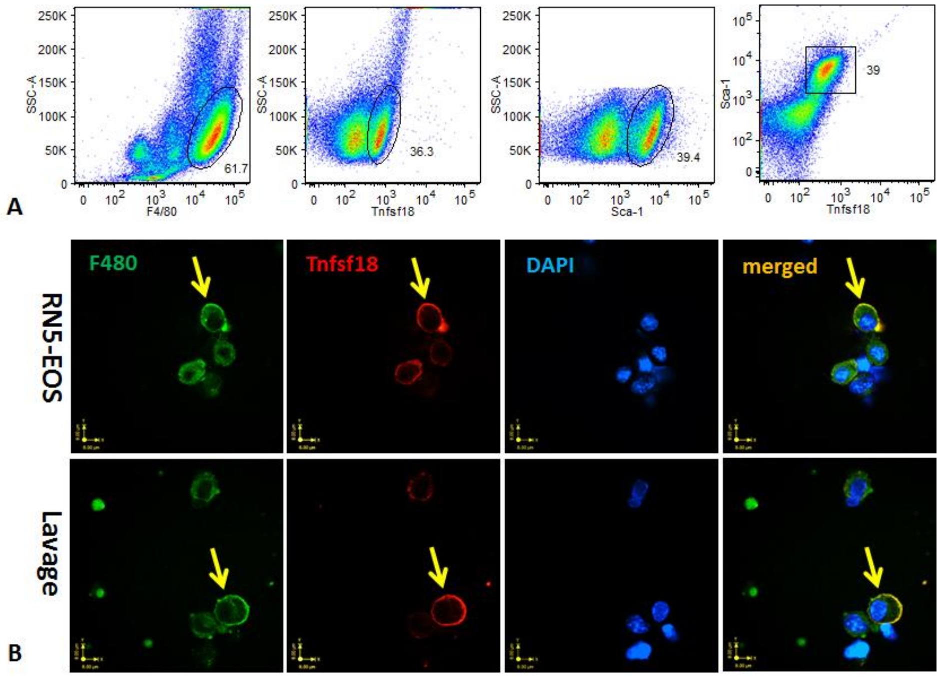

***Supplementary figure 3. In vitro assay of tumor-specific T cell proliferation determined by flow cytometry.*** *Compared with naïve mice, splenocytes derived from RN5 tumor-bearing mice had a significantly higher rate of T cell proliferation (evidenced by Ki67) after overnight treatment with or without pulsation of lysates. Pulsation with RN5-EOS-Puro2 (MSC-enriched) lysates induced similar T cell activation as bulk RN5 cell lysates (A); Pulsation with lysates resulted in significant increase of T cell proliferation compared with the splenocytes of naïve (N) mice (B).*

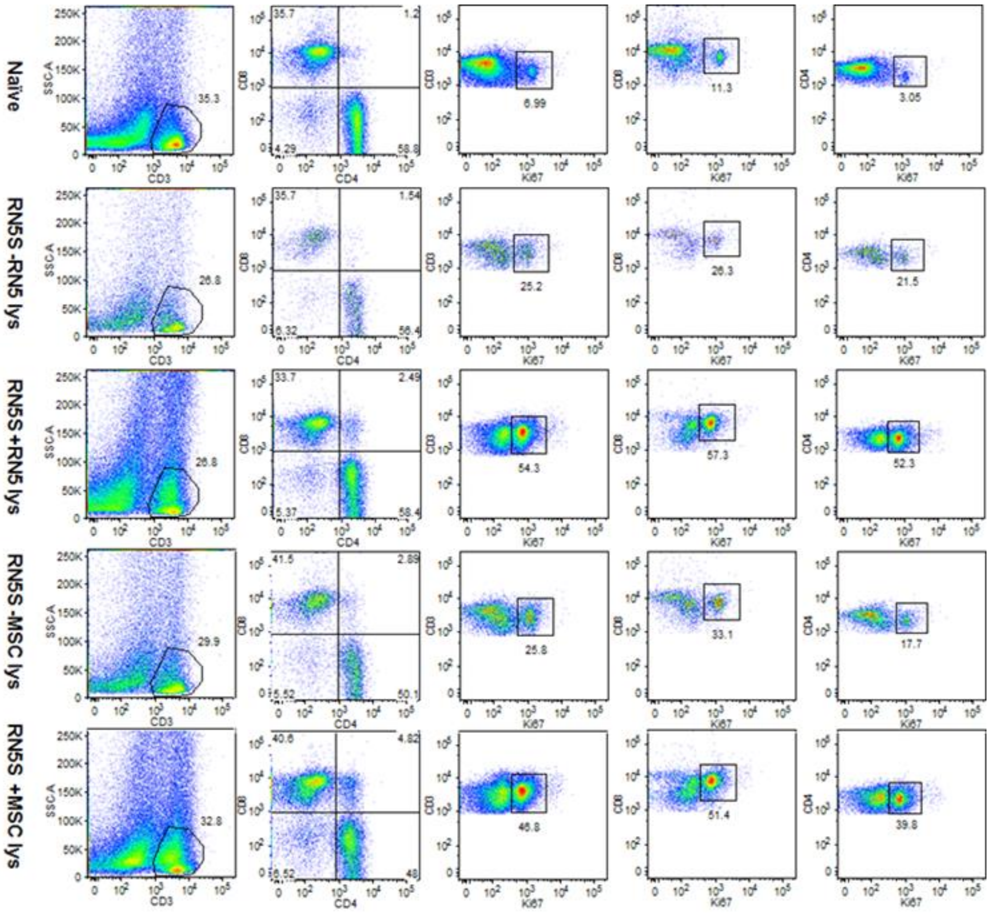


**A**

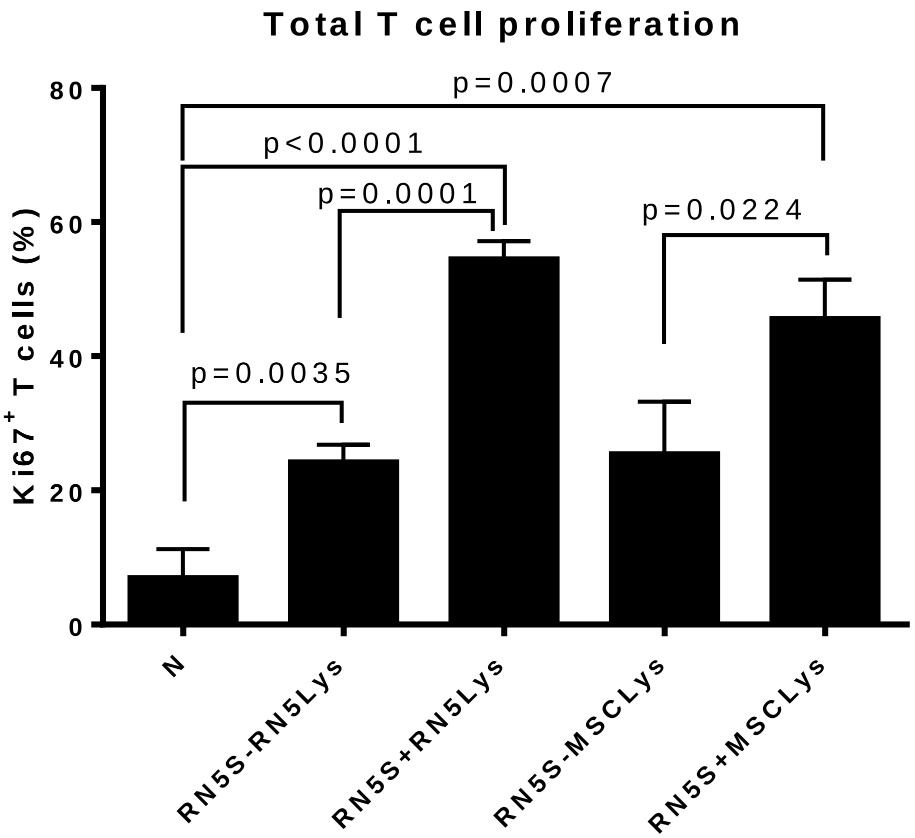
Total T cell proliferation
p=0.0007
p<0.0001
p=0.0001
p=0.0224
p=0.0035
Ki67+ T cells (%)
80
60
40
20
0
N
RN5S-RN5Lys
RN5S+RN5Lys
RN5S-MSCLys
RN5S+MSCLys

**B**

**Supplementary Table 1** The selected gene list with 2 or more fold change in comparison with untreated RN5 cells

| | | p-value NoRx/Cis | Fold-Change NoRx/Cis | p-value NoRx/RT | Fold-Change NoRx/RT | p-value NoRx/MSC | Fold-Change NoRx/MSC |
|---|---|---|---|---|---|---|---|
| **Gene Symbol** | RefSeq | NoRx/Cis | NoRx/Cis | NoRx/RT | NoRx/RT | NoRx/MSC | NoRx/MSC |
| **Nppb** | NM_001287348 | 5.08E-11 | 3.46728 | 2.30E-10 | 2.79787 | 6.41E-11 | 3.34607 |
| **Serpinb9b** | NM_011452 | 2.11E-11 | 7.67504 | 4.44E-09 | 2.83068 | 5.74E-12 | 11.0156 |
| **A530046M15Rik** | NR_046131 | 3.59E-10 | 6.20579 | 1.92E-08 | 3.0176 | 4.53E-08 | 2.69278 |
| **Ccl5** | NM_013653 | 4.35E-11 | 5.94997 | 3.15E-08 | 2.17664 | 4.24E-10 | 3.81883 |
| **Gm6502** | XM_006535306 | 3.84E-07 | 2.09144 | 4.12E-08 | 2.6695 | 3.77E-07 | 2.09516 |
| **Gm6502** | XM_006535306 | 5.46E-07 | 2.05338 | 5.97E-08 | 2.60074 | 3.17E-07 | 2.16338 |
| **Fam71f1** | NM_001289663 | 2.13E-10 | 4.37838 | 6.99E-08 | 2.03488 | 8.71E-09 | 2.52306 |
| **Ly6a** | NM_001271416 | 5.71E-09 | 2.65776 | 7.65E-08 | 2.02012 | 1.18E-08 | 2.44015 |
| **Tnfsf18** | NM_183391 | 1.23E-08 | 2.73531 | 7.66E-08 | 2.22059 | 2.43E-10 | 5.2116 |
| **Csf2** | NM_009969 | 2.97E-09 | 16.6869 | 8.47E-08 | 6.29559 | 6.88E-06 | 2.8256 |
| **Sprr1a** | NM_009264 | 1.83E-09 | 5.27875 | 1.48E-07 | 2.59303 | 2.72E-08 | 3.26421 |
| **LOC625240** | XM_889788 | 1.24E-06 | 2.534 | 2.17E-07 | 3.20489 | 6.67E-07 | 2.73901 |
| **Nr4a1** | NM_010444 | 9.09E-09 | -3.09293 | 2.18E-07 | -2.12424 | 4.73E-10 | -5.15002 |
| **Gm6509** | ENSMUST00000098286 | 1.23E-06 | 2.3137 | 2.45E-07 | 2.80923 | 6.85E-07 | 2.47126 |
| **Spp1** | NM_001204201 | 5.81E-09 | 3.66073 | 6.17E-07 | 2.0451 | 1.66E-07 | 2.3338 |
| **Ngf** | NM_013609 | 2.31E-08 | 3.53783 | 6.70E-07 | 2.27235 | 3.79E-07 | -2.41958 |
| **Igfbp5** | NM_010518 | 1.46E-09 | -6.62552 | 7.52E-07 | -2.34755 | 7.72E-07 | -2.34047 |
| **Ccl17** | NM_011332 | 1.07E-09 | 12.4757 | 8.79E-07 | 2.92424 | 1.77E-06 | 2.66352 |
| **Pglyrp3** | NM_207247 | 3.09E-08 | 3.07909 | 1.13E-06 | 2.03019 | 5.24E-08 | 2.86256 |
| **Gm7982** | XM_006535314 | 1.70E-05 | 2.00772 | 1.61E-06 | 2.595 | 9.86E-06 | 2.11711 |
| **n/a** | --- | 3.01E-05 | 2.79469 | 2.65E-06 | 4.15228 | 3.61E-06 | 3.92296 |
| **Gm7982** | XM_006535314 | 2.29E-05 | 2.07099 | 2.94E-06 | 2.60858 | 2.18E-05 | 2.08136 |
| **Gm6502** | XM_006535306 | 1.40E-05 | 2.6015 | 3.15E-06 | 3.20966 | 1.80E-05 | 2.51963 |
| **Gm26870** | ENSMUST00000181957 | 2.25E-06 | 5.59193 | 4.82E-06 | 4.74211 | 2.06E-08 | 23.272 |
| **Btg2** | NM_007570 | 2.68E-08 | -4.24088 | 6.20E-06 | -2.04276 | 1.84E-07 | -3.09487 |
| **Mmp13** | NM_008607 | 2.84E-07 | 3.24736 | 9.89E-06 | 2.09489 | 1.07E-05 | 2.07884 |
| **Prl2c2** | NM_031191 | 1.03E-05 | 3.60772 | 3.07E-05 | 3.0223 | 4.98E-08 | 13.0249 |
| **Crabp2** | NM_007759 | 8.57E-07 | 3.3316 | 3.54E-05 | 2.08081 | 1.37E-06 | 3.10186 |
| **Gm7963** | ENSMUST00000165454 | 0.000327 | 2.80353 | 4.50E-05 | 3.94076 | 6.01E-05 | 3.73136 |
| **n/a** | --- | 0.000178 | -4.2179 | 0.000103 | 4.7464 | 6.51E-06 | 9.73731 |
| **Gm19654** | AK051476 | 5.41E-07 | -4.43468 | 0.00011 | -2.07142 | 1.51E-05 | -2.61073 |
| **Gm22188** | ENSMUST00000179875 | 0.00026 | 4.56155 | 0.000184 | 4.93439 | 0.00054 | 3.89279 |
| **LOC102635721** | XR_382369 | 4.68E-06 | 3.20511 | 0.000196 | 2.0036 | 1.04E-05 | 2.84972 |
| **Prl2c3** | NM_011118 | 5.34E-05 | 2.39988 | 0.000227 | 2.0375 | 8.57E-08 | 7.74848 |
| **Gm25225** | ENSMUST00000158197 | 0.000108 | -3.04738 | 0.000402 | -2.50503 | 0.00032 | -2.58729 |
| **Gm24382** | ENSMUST00000122698 | 1.54E-05 | -3.76435 | 0.000611 | -2.18732 | 0.000173 | -2.57417 |
| **Ly6c2** | NM_001099217 | 2.03E-05 | 3.70679 | 0.000625 | 2.22595 | 0.000231 | 2.53427 |
| **LOC624931** | XM_006536388 | 0.002918 | 2.50379 | 0.000905 | 3.04692 | 0.003713 | 2.41022 |
| **Gm23862** | ENSMUST00000180342 | 0.000467 | 2.79083 | 0.001033 | 2.4768 | 0.00196 | 2.26291 |
| **n/a** | --- | 0.000146 | -3.26686 | 0.001702 | -2.25179 | 9.71E-06 | -5.60768 |
| **LOC625240** | XM_889788 | 0.006632 | 2.30924 | 0.001945 | 2.832 | 0.0067 | 2.30549 |

**Supplementary table 2** Selected genes of potential murine mesothelioma stem cells overlapped with human gene signature profiling (GSEA)

| Entrez Gene ID | Gene Symbol | Gene Description |
|---|---|---|
| **4803** | NGF | Nerve growth factor (beta polypeptide) |
| **4879** | NPPB | Natriuretic peptide B |
| **6361** | CCL17 | Chemokine (C-C motif) ligand 17 |
| **1437** | CSF2 | Colony stimulating factor 2 (granulocyte-macrophage) |
| **6696** | SPP1 | Secreted phosphoprotein 1 |
| **1382** | CRABP2 | Cellular retinoic acid binding protein 2 |
| **6698** | SPRR1A | Small proline-rich protein 1A |
| **6352** | CCL5 | Chemokine (C-C motif) ligand 5 |
| **8995** | TNFSF18 | Tumor necrosis factor (ligand) superfamily, member 18 |
| **4322** | MMP13 | Matrix metallopeptidase 13 (collagenase 3) |
| **114771** | PGLYRP3 | Peptidoglycan recognition protein 3 |
| **84691** | FAM71F1 | Family with sequence similarity 71, member F1 |
| **84875** | PARP10 | Poly (ADP-ribose) polymerase family, member 10 |

**Supplementary table 3** GO signaling pathways associated with the selected 41 genes

| **Pathway** | **FDR*** |
|---|---|
| GO:0090066_regulation_of_anatomical_structure_size | 0.006421 |
| GO:0048588_developmental_cell_growth | 0.010192 |
| GO:0009617_response_to_bacterium | 0.01004 |
| GO:0032846_positive_regulation_of_homeostatic_process | 0.007571 |
| GO:0043069_negative_regulation_of_programmed_cell_death | 0.0055 |
| GO:0048545_response_to_steroid_hormone_stimulus | 0.005667 |
| GO:0060548_negative_regulation_of_cell_death | 0.00815 |

*FDR: False discovery rate. FDR<0.01
